# Comparative analysis of large language models on rare disease identification

**DOI:** 10.1186/s13023-025-03656-w

**Published:** 2025-04-01

**Authors:** Guangyu Ao, Min Chen, Jing Li, Huibing Nie, Lei Zhang, Zejun Chen

**Affiliations:** 1 Department of Nephrology, Chengdu First People’s Hospital, No.18 Wanxiang North Road, High-tech District, Chengdu, 610095 Sichuan China; 2 Chengdu University of Traditional Chinese Medicine, Chengdu, Sichuan China; 3 Sichuan Provincial Geriatrics Clinical Medical Research Center, Chengdu, China

**Keywords:** Rare disease, Large language models, Diagnostic accuracy

## Abstract

Diagnosing rare diseases is challenging due to their low prevalence, diverse presentations, and limited recognition, often leading to diagnostic delays and errors. This study evaluates the effectiveness of multiple large language models (LLMs) in identifying rare diseases, comparing their performance with that of human physicians using real clinical cases. We analyzed 152 rare disease cases from the Chinese Medical Case Repository using four LLMs: ChatGPT-4o, Claude 3.5 Sonnet, Gemini Advanced, and Llama 3.1 405B. Overall, the LLMs performed better than human physicians, and Claude 3.5 Sonnet achieved the highest accuracy at 78.9%, significantly surpassing the accuracy of human physicians, which was 26.3%. These findings suggest that LLMs can improve rare disease diagnosis and serve as valuable tools in clinical settings, particularly in regions with limited resources. However, further validation and careful consideration of ethical and privacy issues are necessary for their effective integration into medical practice.

**Supplementary Information:**

The online version contains supplementary material available at 10.1186/s13023-025-03656-w.

## Introduction

The diagnosis of rare diseases presents significant challenges due to their low prevalence, diverse syndromic presentations, limited clinical recognition, and the absence of reliable monitoring tools. These challenges frequently lead to diagnostic delays or misdiagnoses, worsening symptoms, causing additional complications, and ultimately leading to poorer outcomes [1]. Over 350 million people worldwide are affected, leading to significant economic burdens and adverse outcomes [2]. Patients with rare diseases often face prolonged diagnostic processes, frequent hospitalizations and long-term complications due to the limited effectiveness of treatments. Thus, it is essential to develop tools for early diagnosis, improved treatment effectiveness, and better condition monitoring to enhance care quality and reduce costs.

Recent advancements in artificial intelligence, especially large language models (LLMs), provide promising solutions for rare diseases diagnosis [3]. Trained on billions of words from articles, books, and other medical literature, LLMs can process and interpret complex patterns in language and data [4]. By integrating comprehensive clinical information, these models utilize their extensive knowledge base to identify rare diseases and suggest potential diagnoses. However, the comparative performance of these LLMs in rare disease diagnosis has not been systematically evaluated. ChatGPT-4o by OpenAI, Claude 3.5 Sonnet by Anthropic, Gemini Advanced by Google DeepMind, and Llama 3.1 405B by Meta are four widely used LLMs.. In this study, we evaluated the diagnostic performance of these LLMs in identifying rare diseases and compared their performance with those of human physicians using real clinical cases.

## Methods

We obtained rare disease case data from the Chinese Medical Case Repository (CMCR). Each case was confirmed as a rare disease by its inclusion in the NIH's Genetic and Rare Diseases Information Center (GARD) database [5] or the Chinese Rare Diseases List (CRDL) [6]. To better simulate real-world clinical diagnostic challenges, we carefully selected the data to include and exclude specific information from patient records. we excluded pathognomonic indicators such as genetic test results, tissue biopsies and other characteristic pathological markers that could make the diagnosis obvious. Retained information included clinical history, physical examination findings, demographic details, symptom descriptions, and routine laboratory data (e.g., complete blood count, basic metabolic panels, urinalysis). These data elements reflect the types of information typically available in general hospital settings during initial evaluations. Importantly, only records available prior to the patient's first confirmed diagnosis with the rare disease were used for analysis. This approach was designed to replicate the diagnostic conditions that physicians frequently face, where definitive markers are absent and diagnosis must rely primarily on clinical reasoning and basic available data.

The cases were analyzed using four LLMs: ChatGPT-4o, Claude 3.5 Sonnet, Gemini Advanced, and Llama 3.1 405B. Based on the provided case information, each model generated the top five most likely diagnoses, ranking them by probability. Diagnostic performance was evaluated using two key metrics: accuracy and weighted accuracy. Accuracy measured the proportion of correct diagnoses, while weighted accuracy took into account both the correctness of the diagnoses and their ranking order. Weights were assigned as follows: 1st place: 5, 2nd place: 4, 3rd place: 3, 4th place: 2, 5th place: 1. The weighted accuracy was calculated as

$${\text{Weighted}}\;{\text{Accuracy }}=\sum\limits_{{{\text{i = 1}}}}^{{\text{5}}} {\left( {\frac{{{\text{correct}}\;{\text{diagoses}}\;{\text{ at}}\;{\text{ rank }}\;{\text{i}}}}{{{\text{total }}\;{\text{cases}}}} \times {\text{Weight}}_{{\text{i}}} } \right)}$$

For human physician evaluation, we initially recruited three chief physicians from the department of nephrology, each with over 15 years of clinical experience. The physicians were provided with the same clinical information given to the LLMs and were asked to provide five possible diagnoses for each case. Due to the complexity of the rare disease cases, two physicians withdrew from the study before completion, only one physician completed the evaluation of all 152 cases. For some challenging cases, the physician was unable to generate five diagnostic hypotheses. Therefore, we focused our analysis on diagnostic accuracy alone, defined as whether the correct diagnosis appeared among the physician's proposed diagnoses.

Statistical analyses were performed using Python 3.7.0, with 95% CIs derived from binomial distributions and pairwise comparisons conducted via two-tailed *t* tests (significance: P < 0.05).

## Results

Our study included 152 cases representing 66 distinct rare diseases, encompassing a diverse range of conditions including metabolic disorders (e.g., phenylketonuria, biotinidase deficiency, carnitine deficiency), genetic disorders (e.g., Alport syndrome, Fabry disease, Marfan syndrome), autoimmune conditions (e.g., autoimmune encephalitis, autoimmune hypophysitis), and neurological disorders (e.g., amyotrophic lateral sclerosis, multiple sclerosis, spinal muscular atrophy). A complete list of all rare diseases included in this study is provided in Supplementary Table S1.

**Fig. 1:**
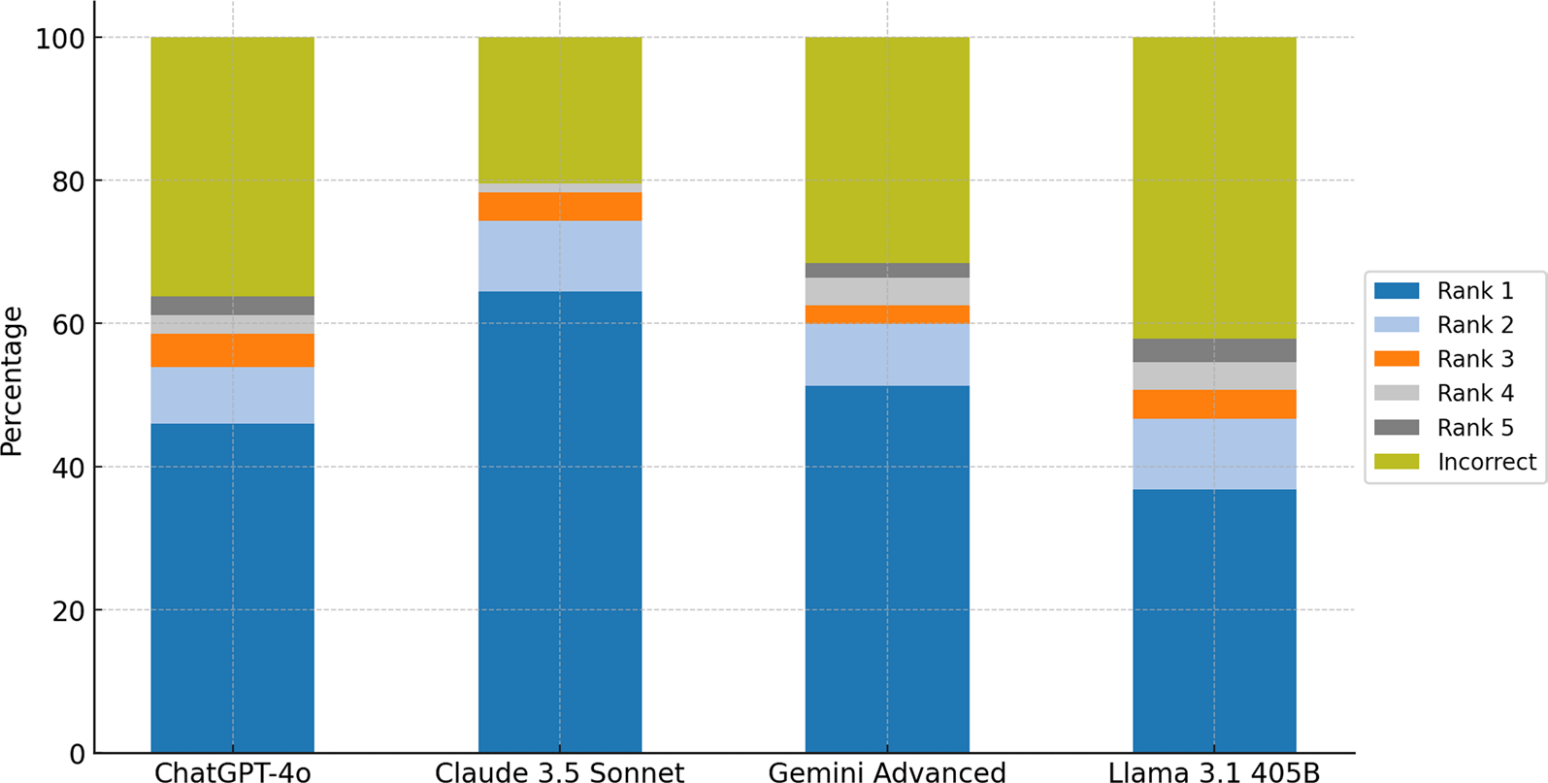
Distribution of diagnostic accuracy and ranking among LLMs

Among the LLMs evaluated, Claude 3.5 Sonnet demonstrated the highest accuracy at 78.9% (95% CI, 71.9–84.9%), showing a significant higher performance compared to the other models: Gemini Advanced achieved 67.8% accuracy (95% CI, 60.4–74.5%), ChatGPT-4o reached 63.2% (95% CI, 55.4–70.6%), and Llama 3.1 405B showed 57.2% accuracy (95% CI, 49.5–64.6%) (Fig. 1). In comparison, human physicians had a considerably lower accuracy rate of 26.3% (95% CI, 20.0–33.6%). Pairwise comparisons between Claude 3.5 Sonnet and other models were all statistically significant (*P* < 0.05). The ranking distribution for each LLM is summarized in Table 1.

**Table 1:** Diagnostic accuracy and ranking distribution

Model	Correct					Incorrect
	Rank 1	Rank 2	Rank 3	Rank 4	Rank 5	
Chatgpt-4o	46.05%	7.24%	4.61%	2.63%	2.63%	36.84%
Claude 3.5 Sonnet	64.47%	9.21%	3.95%	1.32%	0.00%	21.05%
Gemini Advanced	51.32%	7.89%	2.63%	3.95%	1.97%	32.24%
Llama 3.1 405B	36.84%	9.21%	3.95%	3.95%	3.29%	42.76%

**Fig. 2:**
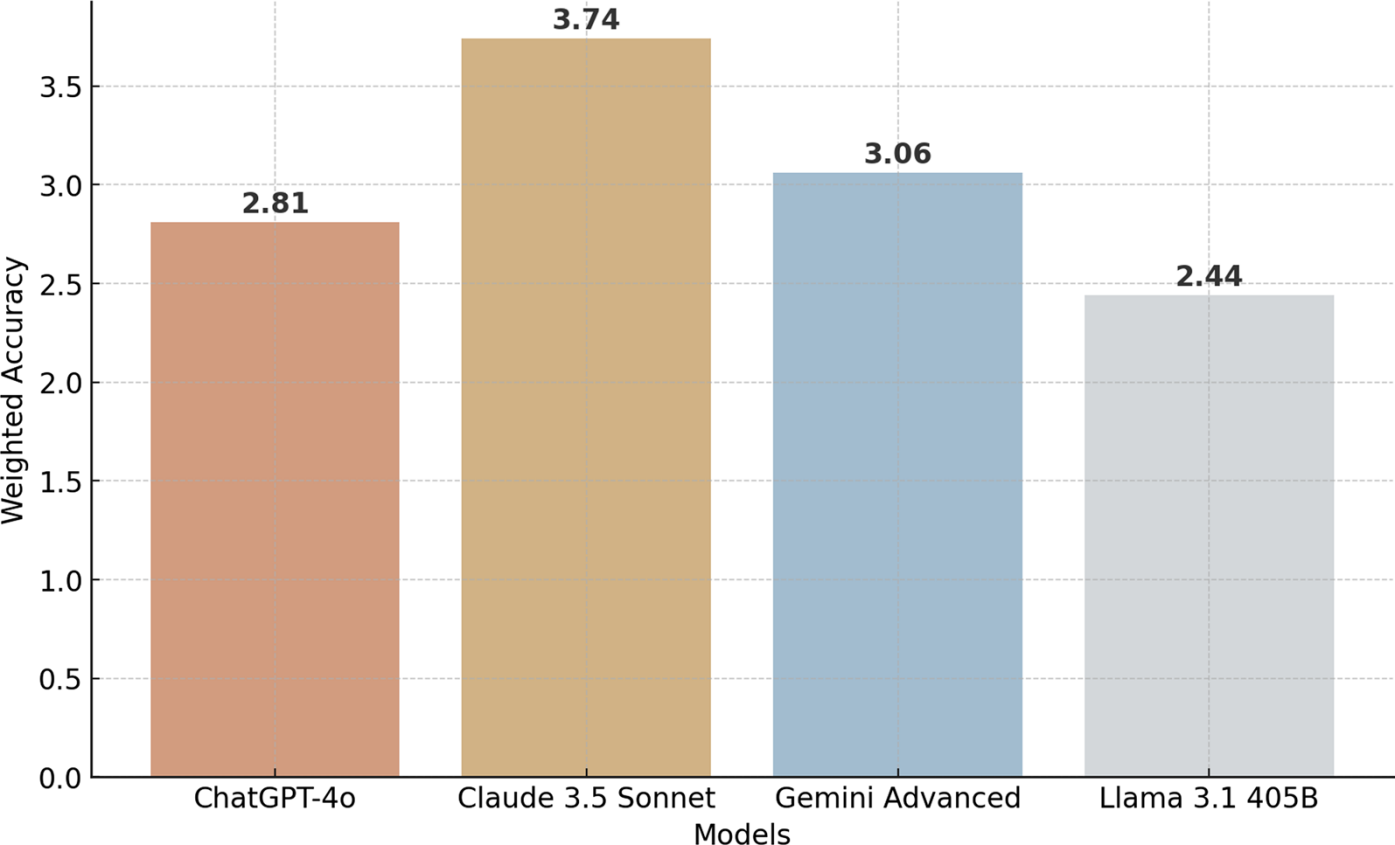
Weighted accuracy of LLMs in rare disease identification

Weighted accuracy scores, which account for both the correctness and the ranking of diagnoses, showed that Claude 3.5 Sonnet had the highest score at 3.74, followed by Gemini Advanced at 3.06, ChatGPT-4o at 2.81, and Llama 3.1 405B at 2.44 (Fig. 2).

## Discussion

Diagnosing rare diseases continues to be a significant challenge in clinical practice. Our study demonstrates that the four evaluated LLMs all surpassed human medical professional in diagnostic accuracy, underscoring their potential as valuable tools for clinical decision-making. Among these LLMs, Claude 3.5 Sonnet exhibited the highest performance in diagnosing complex and rare diseases, achieving superior accuracy compared to the other models. However, as these models are continuously updated, their relative performance may change over time.

A key innovation of our study lies in the use of real-world diagnostic scenarios without definitive disease markers. By relying solely on clinical histories, physical examinations, and routine laboratory data, our study closely simulates the challenges physicians routinely face in daily practice. The relatively limited performance of human physicians reflects the challenges in diagnosing rare diseases. Clinical diagnostic expertise requires extensive accumulation of practical experience. However, the low prevalence of rare diseases severely limits such opportunities. Unlike human physicians, whose diagnostic capabilities are limited by individual clinical experience, LLMs can analyze patterns across millions of documented cases, highlighting their potential as diagnostic aids, especially in regions where expertise in rare diseases is limited.

LLMs have shown significant potential in the medical field, including medical writing, literature searches, and responding to patient inquiries [7]. Previous studies have shown that commercial LLMs, such as GPT-4, are increasingly valuable for medical question answering and clinical decision support tasks [8]. With their extensive knowledge bases and advanced training, these LLMs can significantly enhance the diagnostic capabilities of physicians, providing valuable support in medical decision-making [9]. This potential is particularly crucial for diagnosing complex and rare conditions, which are challenging due to their low prevalence and diverse clinical presentations.

LLMs can act as effective diagnostic aids, particularly in regions with limited medical resources and a shortage of experienced clinicians. However, integrating LLMs into clinical practice requires caution, as their effectiveness may vary across different clinical settings. Additionally, ethical considerations and data privacy concerns require careful attention [10]. Consequently, open-source LLMs present a viable alternative, offering more transparent training processes and improved human oversight [7]. In our study, Llama 3.1 405B, an open-source LLM, showed promising initial performance. Nevertheless, further validation and refinement remain crucial to ensuring the safe and effective application of these models in medicine.

Claude 3.5 Sonnet’s superior performance in our study is consistent with findings from previous research. Studies have shown that Claude excels in tasks requiring comprehensive understanding and context-aware reasoning across various medical domains [11, 12]. In particular, its ability to generate accurate and reliable medical recommendations has been documented in comparative evaluations, where it consistently achieved high scores for accuracy and comprehensiveness across different clinical scenarios[12, 13]. These strengths likely contributed to its success in diagnosing rare diseases in our study. However, as LLMs are being rapidly updated, their relative performance may shift over time, necessitating ongoing evaluation to ensure their effectiveness in clinical applications.

This study has several limitations. First, the predominant use of Chinese case sources may introduce geographical bias. Second, the use of retrospective case report may not fully reflect the complexities of real-time clinical decision-making. Third, models were instructed to provide only a ranked list of up to five most likely diagnoses without probability estimates may have oversimplified the evaluation of diagnostic performance. Fourth, the evaluation of human diagnostic performance was limited by the small number of participating physicians and their singular specialty background, which may not fully represent the broader spectrum of clinical diagnostic capabilities. Finally, the study was limited to four commonly used LLMs.

## Supplementary Information

Additional file 1.

## Data Availability

The data supporting the findings of this study are available upon reasonable request from the corresponding author.
