# Supplementary material for: Comparative analysis of large language models on rare disease identification
Source: Orphanet J Rare Dis. 2025 Apr 1;20:150. doi: 10.1186/s13023-025-03656-w (PMC11959745; doi:10.1186/s13023-025-03656-w)
Supplement: Supplementary file 1 — Additional file 1. [file 13023_2025_3656_MOESM1_ESM.docx]

**Supplementary Table S1: Complete List of Rare Diseases Included in This Study**

| **Number** | **Rare Disease** |
| --- | --- |
| 1 | 21-Hydroxylase Deficiency |
| 2 | Albinism |
| 3 | Alport Syndrome |
| 4 | Amyotrophic Lateral Sclerosis |
| 5 | Autoimmune Encephalitis |
| 6 | Autoimmune Hypophysitis |
| 7 | β-Ketothiolase Deficiency |
| 8 | Biotinidase Deficiency |
| 9 | Primary Carnitine Deficiency |
| 10 | Castleman Disease |
| 11 | Charcot-Marie-Tooth Disease |
| 12 | Citrullinemia |
| 13 | Congenital Myasthenic Syndromes |
| 14 | Erdheim-Chester Disease |
| 15 | Fabry Disease |
| 16 | Familial Mediterranean Fever |
| 17 | Fanconi Anemia |
| 18 | Galactosemia |
| 19 | Gaucher Disease |
| 20 | Gitelman Syndrome |
| 21 | Hemophilia |
| 22 | Wilson's Disease |
| 23 | Hereditary Angioedema |
| 24 | Hereditary Spastic Paraplegia |
| 25 | Homocystinuria |
| 26 | Hyperphenylalaninemia |
| 27 | Idiopathic Pulmonary Arterial Hypertension |
| 28 | Idiopathic Pulmonary Fibrosis |
| 29 | IgG4-Related Disease |
| 30 | Isovaleric Acidemia |
| 31 | Kallmann Syndrome |
| 32 | Langerhans Cell Histiocytosis |
| 33 | Lymphangioleiomyomatosis |
| 34 | Marfan Syndrome |
| 35 | Medium-Chain Acyl-CoA Dehydrogenase Deficiency |
| 36 | Methylmalonic Acidemia |
| 37 | Mitochondrial Encephalomyopathy |
| 38 | Mucopolysaccharidosis |
| 39 | Multifocal Motor Neuropathy |
| 40 | Multiple Acyl-CoA Dehydrogenase Deficiency |
| 41 | Multiple Sclerosis |
| 42 | Multiple System Atrophy |
| 43 | Neuromyelitis Optica |
| 44 | Niemann-Pick Disease |
| 45 | Noonan Syndrome |
| 46 | Ornithine Transcarbamylase Deficiency |
| 47 | Paroxysmal Nocturnal Hemoglobinuria |
| 48 | Peutz-Jeghers Syndrome |
| 49 | Phenylketonuria |
| 50 | POEMS Syndrome |
| 51 | Porphyria |
| 52 | Prader-Willi Syndrome |
| 53 | Progressive Familial Intrahepatic Cholestasis |
| 54 | Progressive Muscular Dystrophy |
| 55 | Propionic Acidemia |
| 56 | Pulmonary Alveolar Proteinosis |
| 57 | Retinitis Pigmentosa |
| 58 | Silver-Russell Syndrome |
| 59 | Sitosterolemia |
| 60 | Spinal Muscular Atrophy |
| 61 | Spinocerebellar Ataxia |
| 62 | Systemic Sclerosis |
| 63 | Tetrahydrobiopterin Deficiency |
| 64 | Tuberous Sclerosis |
| 65 | Very-Long-Chain Acyl-CoA Dehydrogenase Deficiency |
| 66 | X-Linked Adrenoleukodystrophy |
